# Optimization of Ultrasonic Enzyme-Assisted Extraction for the Recovery of Phenolic Compounds and Soluble Solids from Apple Pomace

**DOI:** 10.3390/foods15010098

**Published:** 2025-12-29

**Authors:** Violeta Nour

**Affiliations:** Department of Horticulture & Food Science, University of Craiova, 13 AI Cuza Street, 200585 Craiova, Romania; violeta.nour@edu.ucv.ro

**Keywords:** apple by-products, up-cycling, flavonoids, optimization, phenolic acids, process parameters, sustainability

## Abstract

Apple pomace is a significant by-product of the juice processing industry and a rich source of bioactive compounds; however, its potential as a valuable resource is currently largely untapped. In this work, the ultrasound–enzyme-assisted extraction (UEAE) was evaluated as an alternative method for the extraction of phenolic compounds and soluble solids from apple pomace. For this purpose, an optimization study was carried out using a Box–Behnken factorial design combined with the response surface methodology to assess the influence of enzyme/substrate ratio (0–10% *v*/*w*), extraction time (1–5 h) and temperature (25–55 °C) on three response variables: total phenolic content, DPPH radical scavenging activity and soluble solids content of the extracts. In addition, the phenolic profile of the extracts was also investigated. According to the model, DPPH radical scavenging activity will record the maximum value (0.69 mmol Trolox/L) for a 10% enzyme/substrate ratio, at 42 °C and 1 h extraction time. Extraction with an enzyme/substrate ratio of 8.5% at 41 °C for 1 h gave the highest retrieval of soluble solids content (4.1%) in the extracts. Based on HPLC results, chlorogenic acid, caffeic acid, rutin, and epicatechin were the predominant polyphenols in the extracts. The results confirmed the great potential of apple pomace as an economical source of bioactive compounds, and UEAE enhanced the recovery of phenolic compounds and soluble solids from this underutilized by-product.

## 1. Introduction

With a worldwide production of 95.8 million tonnes in 2022, according to the Food and Agriculture Organization [1], apples (*Malus domestica*) are one of the most widely consumed fruits on the planet. Approximately 70–75% of apples are eaten raw, while 25–30% are used to prepare various products like juice, wine, purees, jams, and dried apple products [2,3]. Apple juice is the most in-demand processed product, accounting for about 65% of the total amount of apples that are processed [4]. During apple juice production, approximately 75% of the apple’s fresh weight is converted to juice, with the remaining 25% becoming the solid food waste known as pomace [5,6].

Worldwide juice production annually generates several million metric tons of pomace. Although considered a safe livestock feed additive, due to its low pesticide concentrations, the poor protein content makes apple pomace a low-quality feed for animals [7,8,9]. Managing apple pomace is costly due to the pollution risks of composting, which include the production of greenhouse gases, the risk of spreading disease, and groundwater contamination [10]. Moreover, its high moisture content (>70%) makes apple pomace highly susceptible to unpredictable fermentation, further complicating its disposal [9,11].

Apple pomace resulting from apple juice processing consists of approximately 95% skins and flesh, 2–4% seeds, and 1% stems [3,9,12]. Apple pomace is a rich source of health-promoting nutrients and bioactive compounds, including minerals, dietary fiber, polyphenols, and ursolic acid [13], suggesting its potential to be used in developing dietary supplements and functional foods [8,14]. The phenolic fraction, which is mainly found in apple peels, contains hydroxycinnamic acids, flavan-3-ols, flavonols, dihydrochalcones, anthocyanins, and hydroxybenzoic acids [15,16]. Preclinical studies showed that apple pomace and its extracts benefit gastrointestinal function, antioxidant status, and lipid metabolism, which positively impacts metabolic disorders like hyperglycemia and insulin resistance [8]. At present, apple pomace is underutilized, with a low rate of recovery and reuse; however, the large volume of pomace presents both an economic opportunity and a waste management challenge, as its commercial applications can be highly profitable [2]. The great potential of apple pomace has raised the need for developing ways to use it effectively in various applications, to improve economic efficiency and contribute to a circular economy. This approach is a key component of achieving sustainable consumption and production patterns, one of the 17 sustainable development goals (SDG12) stipulated in the United Nations 2030 Agenda [17].

In addition to its use in fermentation, apple pomace can be valorized by the extraction of compounds with potential health-protecting capacities, like polyphenols, dietary fibers, and pectin [18]. A growing global movement is focused on the recovery of high-value substances from these underutilized resources through efficient, low-cost, and eco-friendly processes [19,20]. Many studies used organic solvents to extract valuable components from apple pomace, making them suitable for non-food products like pharmaceuticals but unsuitable for food applications due to safety concerns [15]. Water is non-toxic, relatively cheap, readily available, and environmentally friendly, being recognized as the most natural and greenest solvent. These make water a suitable solvent for recovering valuable compounds to be used in nutraceuticals and functional foods [21].

Currently, there is growing interest in using green extraction methods to recover bioactive compounds from fruit by-products instead of conventional methods that often rely on hazardous organic solvents [22,23]. To this end, ultrasound (UAE), microwave (MAE), or enzyme-assisted (EAE) extraction may be mentioned as novel and eco-friendly techniques, with high potential to reduce environmental impact by using less energy, less solvent, or safer solvents, while improving the yield and quality of extracted compounds [24,25]. Enzyme-assisted extraction is increasingly popular due to its potential as a green and efficient method for extracting compounds from plant materials [26]. It uses commercially available cellulolytic and pectinolytic enzymes to disrupt plant cell walls and complex molecules, such as glycosylated conjugates, and to release essential oils and phenolic compounds that are often bound, thus improving extraction yields and selectivity while often requiring less energy and solvent than traditional methods [20,27].

EAE has demonstrated promising results in previous studies as a method for efficiently extracting phenolic compounds from blackcurrant [19,28,29], chokeberry [30], bilberry [20], blueberry [31], lingonberry [22], blackberry [32], American cranberry [33], sweet cherry [34], grape [35,36], cerrado cashew apple [37], olive [38] and sea-buckthorn [39] pomaces. The effectiveness of enzyme-assisted extraction is highly dependent on numerous factors, such as substrate type, enzyme concentration, incubation time, and temperature. Therefore, optimizing these variables is critical for a successful and efficient extraction process [30,36]. Combining advanced techniques like microwave, ultrasound, and supercritical fluid extraction with enzymes has been shown to be effective in recovering bioactive compounds from plant matrices [38,40].

Ultrasound-assisted extraction uses ultrasonic cavitation resulting from high-power ultrasonic waves to break down cell walls, thus achieving superior extraction yields in a very short extraction time [21]. Ultrasonic-enzyme-assisted extraction (UEAE) combines ultrasound and enzyme-assisted extraction to improve the efficiency of obtaining natural bioactive compounds from plant materials [41]. Previously, UEAE was shown to be more efficient in extracting phenolic compounds than UAE or EAE alone and to enhance the bioactivity of plant-derived compounds [42,43,44].

To maximize efficiency, the valorization process must be tailored for each plant material because variations in its composition and properties, as well as process parameters, can significantly alter the outcome. Response surface methodology (RSM) combined with factorial designs is a widely used and effective mathematical modeling technique for optimizing multiple-response processes [45]. This approach efficiently analyzes the interactions between multiple factors and their effects on desired outcomes, allowing for the determination of optimal conditions to improve process efficiency [30,33,34,46].

The aim of this work was to develop an efficient aqueous ultrasonic-enzyme-assisted extraction method for the recovery of phenolic compounds and water-soluble components from apple pomace. The Box–Behnken design was employed to identify the optimal extraction conditions (enzyme/substrate ratio, temperature, and extraction time) that yield the highest extraction of phenolic compounds, antioxidant activity, and soluble solids from the apple pomace. In addition, the composition of individual phenolic compounds in the extract was analyzed using high-performance liquid chromatography.

## 2. Materials and Methods

### 2.1. Plant Material and Pomace Preparation

Apples harvested during September 2025 were processed into juice without enzymatic treatment at Ancorvita S.R.L., a small fruit juice producer in Beharca, Dolj county (South-West Oltenia Region, Romania). Three lots of apple pomace (around 3 kg, moisture content = 77.79 ± 0.12% wt.), consisting of peels, residual pulp, and seeds, created on different days as by-products from apple juice processing, were supplied to the laboratory immediately after being obtained. They were sealed in plastic bags, frozen and kept at −18 °C until use, then thawed at 20 °C before drying. The apple by-product samples were dried in a convective tray dryer (Deca + SS Design, Profimatic, Cluj-Napoca, Romania) at 57 °C until their moisture content dropped below 5%. The dried pomace was then finely ground using an electric mill (Bosch TSM6A011W, Bosch, Munich, Germany), then sieved through a 0.3 mm hole size mesh sieve. The resulting powders were sealed in glass jars and stored in the dark until extraction. The moisture content of the apple pomace powder was determined gravimetrically by measuring the weight loss of 2 g of sample after drying at 103 °C until reaching a constant weight (AOAC method 934.01) [47]. Viscozyme ^®^ L was kindly provided by Novozymes (Bagsværd, Denmark). Viscozyme ^®^ L is a multi-enzyme complex with a wide range of carbohydrase activities, including pectinases, cellulases, and hemicellulases, able to efficiently hydrolyze pectin-rich plant biomass to release bioactive compounds [48].

### 2.2. Chemicals

Folin–Ciocalteu reagent (2 N), 6-hydroxy-2,5,7,8-tetramethylchroman-2-carboxylic acid (Trolox, 98% purity), and methanol (HPLC grade) were purchased from Merk (Darmstadt, Germany). Gallic acid, 2,2-diphenyl-1-picrylhydrazyl (DPPH), anhydrous sodium carbonate, and anhydrous sodium acetate were from Sigma-Aldrich (Steinheim, Germany). Caffeic, chlorogenic, ferulic, gallic, *p*-coumaric, syringic, *trans*-cinnamic, and vanillic acids, as well as catechin hydrate, epicatechin, quercetin, and rutin, were purchased as standard substances from Sigma-Aldrich GmbH (Steinheim, Germany).

### 2.3. Experimental Design

The aqueous extraction of phenolic compounds and soluble solids from apple pomace was modeled and optimized using response surface methodology (RSM). The influences of three independent variables, including the enzyme/substrate ratio (*x*_1_) (0–10% *v*/*w*), extraction time (*x*_2_) (1–5 h), and temperature (*x*_3_) (25–55 °C), on three response variables (total phenolic content, DPPH radical scavenging activity, and total soluble solids) were assayed. The models aim to identify the set of process parameters that maximizes the value of the response variables.

A three-level Box–Behnken design with 3 factors and 15 experimental runs was selected to fit a second-order response surface and to optimize the extraction. Statgraphics Centurion XVI software (StatPoint Technologies, Warrenton, VA, USA) was employed to perform RSM.

The following second-order nonlinear quadratic polynomial Equation (1) was utilized:

(1) $$Y=\;\beta_{0}+\sum_{i=1}^{k}\beta_{i}x_{i}+\sum_{i=1}^{k}\beta_{ii}x_{i}^{2}+\sum_{i=1}^{k-1}\sum_{j=2}^{k}\beta_{ij}x_{i}x_{j}$$

where *Y* is the response, *x_i_* and *x_j_* are the variables, *β*_0_ is the intercept, *β_i_*, *β_ii_*, and *β_ij_* are the linear, the quadratic, and the interaction coefficients of variables *i* and *j*, respectively.

Table 1 lists the independent variables and their coded and actual levels based on the Box–Behnken design. The levels of these variables were chosen based on the literature and preliminary studies [20,30,37].

The analysis of variance (ANOVA) was employed to assess the adequacy of the model, while R-squared (*R*^2^) indicated the accuracy between predicted and experimental values. The model was considered significant at a 95% confidence level (*p* < 0.05).

### 2.4. Extraction of Apple Pomace in Water

Water was the chosen solvent for the production of the extracts, considering their future use, as such or in concentrated form, in foods. Briefly, 1.5 g of apple pomace powder was precisely weighed in a 50 mL flask and mixed with 30 mL of distilled water. Enzyme was added to achieve the appropriate enzyme/substrate ratio (0%, 5% and 10% *v*/*w*). The pH was adjusted to 3.5 by adding 1 N HCl or 1 N NaOH. After one minute of shaking, the flasks were immersed in a Bandelin ultrasonic bath (Bandelin, Berlin, Germany) operating at 35 kHz for the required time (1, 3, or 5 h) and at the required temperature (25, 40, or 55 °C), according to the experimental design (Table 2). Initially, the ultrasonic bath was filled with water at the required temperature, then the temperature was kept within ±1 °C of the target by replacing bath water with hot or cold water as needed. At the end of the extraction time, the flasks were immersed for 10 min in a boiling water bath, then rapidly cooled and centrifuged (6000 rpm for 10 min). The supernatants were collected and passed through Whatman No. 1 filter paper. Each extraction was made in duplicate, and the extracts underwent triplicate analysis to determine total phenolic content, DPPH radical scavenging activity, and soluble solids content.

### 2.5. Soluble Solids Content

The total soluble solids content (%) of the extracts was measured using a Hanna digital refractometer (Hanna Instruments, Woonsocket, RI, USA) according to AOAC Method 932.12 [49]. The reported average values were obtained after triplicate analysis.

### 2.6. Total Phenolic Content

The total phenolic content (TPC) was assayed in the extracts by the Folin–Ciocalteu method using gallic acid as a standard, as described by Singleton et al. [50]. Briefly, an aliquot of extract (0.1 mL) was added to 6 mL of distilled water and mixed with 0.5 mL of Folin–Ciocalteu phenol reagent, freshly diluted with water (1:1 *v*/*v*). After 3 min, 1.5 mL of Na_2_CO_3_ solution (20% *w*/*v*) was added, and the mix was made up to 10 mL with distilled water. After standing in a dark place at 40 °C for 30 min, the absorbance was read at 765 nm with a Varian Cary 50 UV spectrophotometer (Varian Co., Palo Alto, CA, USA). The total phenolic content of the extracts was calculated from the regression equation of the calibration curve [y = (x + 0.0272)/0.0011, c = 50–300 mg/L] and expressed as mg gallic acid equivalents (GAE) per liter of extract (mg GAE/L).

### 2.7. DPPH Radical Scavenging Activity

DPPH radical scavenging activity (RSA) was tested according to the procedure of Brand-Williams et al. [51]. The analysis was carried out by reacting 50 μL of pomace extract with 3 mL of 0.004% DPPH methanolic solution. The mixture was shaken and placed in the dark for 30 min at 20 °C, then the absorbance was read at 517 nm against methanol. A control sample was similarly prepared by replacing the pomace extract with methanol. The DPPH radical scavenging activity was calculated as the inhibition percentage of the DPPH radical as follows:

DPPH scavenging activity (%) = [1 − absorbance of the sample/absorbance of the control] × 100.

A calibration curve was prepared (y = x/31.428, c = 0.25–2.5 mmol Trolox/L) using Trolox methanolic solution as a reference standard, and the results were expressed as mmol Trolox/L of extract. The analysis was performed in triplicate for each extract.

### 2.8. Quantification of Phenolic Compounds

Twelve phenolic compounds were assessed in the apple pomace extracts by RP-HPLC on a Finningan Surveyor Plus HPLC chromatographic system (Thermo Electron Corporation, San Jose, CA, USA) according to the method developed by Nour et al. [52]. The separation was performed at 20 °C on a Hypersil Gold C18 column (5 μm, 250 × 4.6 mm) using as mobile phase a mixture of 1% aqueous acetic acid (eluent A) and methanol (eluent B) and the following elution program: gradient from 90% A to 80% A in 20 min, from 80% A to 60% A in 7 min, 60% A during 25 min, from 60% A to 80% A in 5 min and from 80% A to 90% A in 3 min. Three chromatograms were simultaneously recorded at 254, 278, and 300 nm. The concentrations of phenolic compounds were calculated based on the detected peak area using external calibration and expressed as mg per liter of extract.

### 2.9. Statistical Analysis

Statgraphics Centurion XVI.I software (StatPoint Technologies, Warrenton, VA, USA) was used to perform RSM. The extraction experiments were carried out in duplicate, and the results were expressed as mean values ± standard deviation (SD). The results were compared using one-way analysis of variance (ANOVA) followed by the LSD test, and differences at *p*-values below 0.05 were considered statistically significant.

## 3. Results and Discussion

The moisture content of apple pomace powder was 4.71 ± 0.23%. A total phenolic content of 433.82 ± 7.67 mg GAE/100 g and a DPPH radical scavenging activity of 1.28 ± 0.57 mmol Trolox/100 g were found in the apple pomace powder used in the experiments.

The response values of the experimental runs acquired for total phenolic content, DPPH radical scavenging activity, and soluble solids content for the actual levels of process parameters (enzyme/substrate ratio, extraction time, and temperature) are presented in Table 2.

Table 3 presents the coefficients for the quadratic models predicting TPC, RSA, and SSC, along with the corresponding analysis of variance (ANOVA) for each model. This table provides the key statistical information needed to interpret how well each model fits the data and the significance of the model’s components. Moreover, these data are used to understand how the independent variables influence the responses, as positive coefficients indicate synergistic effects and negative coefficients indicate antagonistic effects.

R-squared is a statistical measure of how well the model fits the observed data; a higher R-squared suggests a better fit. In our study, the R-squared values for the three models ranged from 75.83% to 96.98%. These values indicated that the developed models were satisfactory. In addition, ANOVA was used to evaluate the adequacy of the developed models and results.

Since the *p*-value for the regression test was lower than 0.05, the models were considered adequate for response variable prediction.

In order to analyze interactions between variables, 3D response surface graphics have been plotted, representing the relationship between two independent variables and a response and showing how the response changes across different combinations of the two variables.

### 3.1. Optimization of Total Phenolic Content

**Figure 1:**
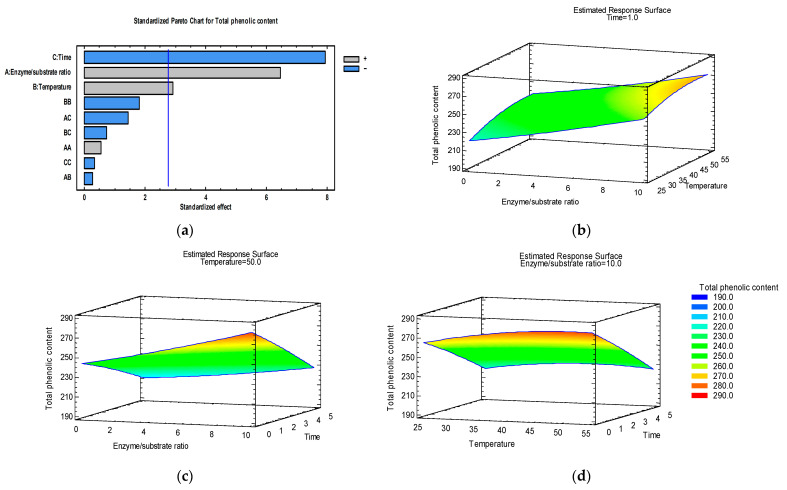
Pareto chart (**a**) and estimated response surface graphics of total phenolic content (mg GAE/L) as a function of temperature and the enzyme/substrate ratio at 1 h extraction time (**b**), enzyme/substrate ratio and time at 50 °C temperature (**c**), and extraction time and temperature at a 10% enzyme/substrate ratio (**d**).

The combination of factors that maximizes total phenolic content was as follows: E/S = 10%, T = 50 °C, and t = 1 h. These process parameters determined an optimal predicted TPC value of 272 mg GAE/L in the apple pomace extract treated with Viscozyme L. By setting the value of each factor to the optimum value, estimated response surface graphics have been plotted (Figure 1b–d).

The Pareto chart (Figure 1a) indicated that total phenolic content was significantly influenced by the linear effect of all process parameters (enzyme/substrate ratio, temperature, and extraction time), as only these factors had *p*-values less than 0.05. The R-squared indicated that the model as fitted explains 75.83% of the variability in total phenolic content. A lack-of-fit test was performed to determine whether the selected model is adequate to describe the observed data, or whether a more complicated model should be used. The test was performed by comparing the variability of the current model residuals to the variability between observations at replicate settings of the factors. Since the *p*-value for lack-of-fit in the ANOVA table (0.2212) was greater than 0.05, the model appeared to be adequate for the observed data at the 95.0% confidence level.

The regression coefficients of the quadratic model for TPC and the Pareto chart indicated that extraction time exerted a negative effect, while enzyme/substrate ratio and temperature exerted a positive effect on TPC. The results (Table 2) demonstrated that by prolonging the extraction time from 1 h to 5 h, TPC values in the extract reduced by 10.84% at 40 °C (E/S = 10%), by 16.83% at 25 °C, and by 19.80% at 55 °C (E/S = 5%).

When evaluating the influence of enzyme–substrate ratio and temperature (Figure 1b) at 1 h extraction time, it was possible to note the increase in the TPC analytical response as the enzyme–substrate ratio increased from 0 to 10%. By adding Viscozyme L at a 10% enzyme/substrate ratio in the ultrasound-assisted extraction at 55 °C for 3 h, total phenolic content increased by 17.93% (from 222.15 mg GAE/L to 262.00 mg GAE/L). Kitrytė et al. [30] achieved increases in the yield of polyphenols from 10.5% (E/S 1% *v*/*w*, 25 °C, pH 5.5, 1 h) to 55.3% (E/S 5.5% *v*/*w*, 40 °C, pH 4.5, 8 h) as compared to the samples without the enzyme.

Regarding the temperature and extraction time (Figure 1d), there was a decrease in the TPC analytical response as the time increased from 1 h to 5 h, and a maximum response at a temperature close to 50 °C. Contrary to our results, Kitrytė et al. [30] found significant synergistic effects between extraction temperature and time in the Viscozyme L-assisted extraction of chokeberry pomace. The optimal conditions for EAE of phenolic compounds from chokeberry pomace using Viscozyme L found in that study were the following: E/S ratio 6% *v*/*w*, temperature 40 °C, extraction time 7 h, and pH 3.5.

The optimal extraction time can emerge from the relative contribution of two outcomes of the enzyme’s activity: (a) the increased disruption of the cell walls resulting in enhanced release of the phenolic compounds; (b) the susceptibility of released phenolic compounds to enzymatic degradation [53]. Cellulases, hemicellulases, and pectinases work together to break down plant cell walls by cleaving different bonds (β-1,4-glycosidic bonds, ester bonds, and pectic galacturonan), thus releasing simpler molecules like oligosaccharides, monosaccharides, and phenolic glycosides [54]. In ultrasonic enzyme-assisted extraction, the ultrasound treatment also contributes to the increase in bioactive content in the extracts, as cavitation promotes a more efficient release of the compounds from their cellular compartments [55]. This phenomenon enhances the mass transfer from the sample to the solvent, allowing for faster and more efficient extraction of compounds even at lower temperatures [29].

In a study comparing different extraction techniques (accelerated solvent extraction, ultrasound-assisted extraction, ultraturrax extraction, and pulsed electric field extraction pre-treatment) for the recovery of phenolic compounds from apple pomace, Pollini et al. [56] found that UAE gave the highest TPC values in the extract. El Kantar et al. [57] enhanced the release of polyphenols from orange peels by combining high-voltage electrical discharges (HVEDs) with enzymatic hydrolysis using Viscozyme^®^ L, showing that this physical pretreatment significantly improved the efficiency of the enzymatic process. Stanek-Wandzel et al. [58] also highlighted the potential of enzyme-assisted extraction to enhance the recovery of polyphenolic compounds and antioxidant activity of the extracts from grape pomace.

### 3.2. Optimization of DPPH Radical Scavenging Activity

**Figure 2:**
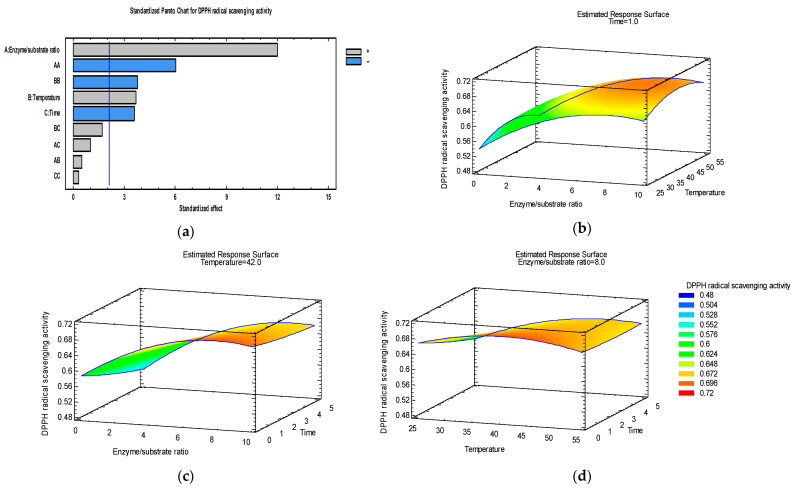
Pareto chart (**a**) and estimated response surface graphics of DPPH radical scavenging activity (mmol Trolox/L) as a function of the enzyme/substrate ratio and temperature at 1 h extraction time (**b**), enzyme/substrate ratio and time at 42 °C temperature (**c**), and extraction time and temperature at an 8% enzyme/substrate ratio (**d**).

Figure 2 presents the response surface graphics which visualize the interaction of enzyme–substrate ratio/temperature (Figure 2b), enzyme–substrate ratio/time (Figure 2c), and temperature/extraction time (Figure 2d) and their influence on the DPPH radical scavenging activity. They were plotted at the optimum value of each process parameter. According to the model, the maximum value of the DPPH radical scavenging activity (0.69 mmol Trolox/L) of the apple pomace extract is reached under the following extraction conditions: temperature = 42 °C, extraction time = 1 h, enzyme/substrate ratio = 8%.

The addition of Viscozyme L at a 10% enzyme/substrate ratio in the ultrasound-assisted extraction at 40 °C during 5 h increased the DPPH radical scavenging activity of the extracts from 0.53 mmol Trolox/L to 0.66 mmol Trolox/L (by 24.52%) (Table 2). Previously, Petrov Ivanković et al. [59] found that Viscozyme^®^ L can be enhanced with 63.4% of the antioxidant activity of the blackcurrant extracts incubated at 50 °C, with shaking, for one hour.

The R-squared indicated that the model as fitted explains 92.16% of the variability in RSA. The antioxidant activity was significantly influenced by the linear effect of all process parameters and by the quadratic effect of enzyme/substrate ratio and temperature.

The Pareto chart (Figure 2a) indicated that extraction time exerted a negative effect, while enzyme/substrate ratio and temperature exerted a positive effect on DPPH radical scavenging activity. Domínguez-Rodríguez et al. [34] also reported that extraction time had a significant negative effect on the DPPH radical scavenging activity of the extracts from cherry pomace. In agreement with our results, Syrpas et al. [20] found that ABTS scavenging activity of the bilberry pomace extracts showed the highest activity after 1 h of enzyme hydrolysis (with Viscozyme L) at 50 °C, followed by a progressive decrease up to 7 h of extraction, while Kapasakalidis et al. [28] reported similar results in a study on enzyme-assisted extraction of blackcurrant pomace using Celluclast. They also reported that enzyme concentration and extraction time significantly influenced the radical-scavenging activity of the water-soluble fractions of bilberry or blackcurrant pomace. Authors reported that at high enzyme concentration, elevated extraction temperature (50 °C), and longer extraction time, the antioxidant activity of the extracts reduced, and attributed these effects to the thermal degradation of phenolic compounds and/or to their altered activity. Damage to other heat-labile antioxidant compounds is also not excluded.

### 3.3. Optimization of Soluble Solids Content

**Figure 3:**
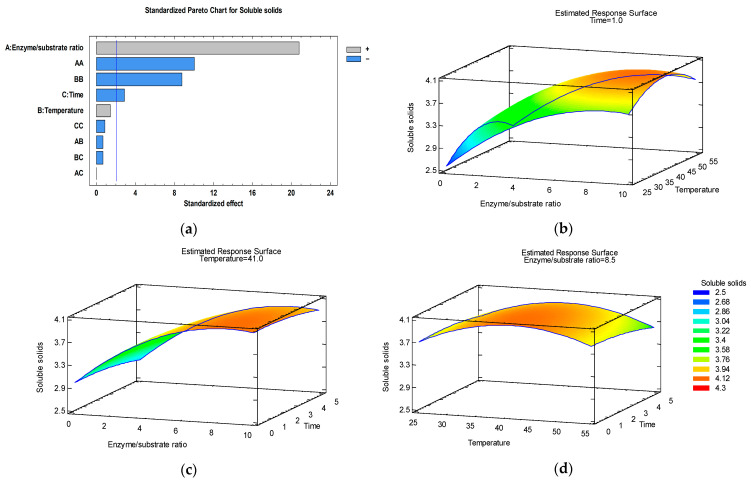
Pareto chart (**a**) and estimated response surface graphics of soluble solids content (%) as a function of temperature and enzyme/substrate ratio at 1 h extraction time (**b**), enzyme/substrate ratio and time at 41 °C temperature (**c**), and extraction time and temperature at an 8.5% enzyme/substrate ratio (**d**).

The maximum value of soluble solids content (SSC = 4.1%) has been reached at the following combination of extraction process parameters: E/S = 8.5%, T = 41 °C, and t = 1 h. As presented in the Pareto chart (Figure 3a), four effects have *p*-values less than 0.05: enzyme/substrate ratio (positive effect) and time (negative effect), as well as the quadratic effect of enzyme/substrate ratio and temperature (negative effects).

Of the three models, this model achieved the highest R-squared (96.98%) and desirability (99.37%), which demonstrated a better model adequacy.

The ultrasound–enzyme-assisted extraction at 40 °C during 1 h increased the soluble solids content by 33.89% (from 2.95% to 3.95%) by adding Viscozyme L at an enzyme/substrate ratio of 10% (Table 2). Davidson et al. [60] also reported that the ultrasound–enzyme-assisted extraction method resulted in higher sugar content in blackberry pomace extract relative to the conventional extraction method.

Therefore, around 4% water-soluble components could be recovered from the apple pomace powder after one hour enzyme- and ultrasound-assisted extraction in water. In large-scale processing, these aqueous extracts could be concentrated by ultrafiltration, evaporation, freeze concentration, reverse osmosis or membrane filtration processes, or by other efficient and sustainable processes combining pasteurization and membrane distillation [61,62,63]. The concentrated apple pomace extracts can be incorporated in bread and sweet bakery products, ciders, and meat products to improve their health-promoting properties and nutritional value [6]. For example, Fernandes et al. [15] increased the polyphenolic content and antioxidant activity of yogurt by adding 3.3% apple pomace extract prepared through hot water extraction. They found that water-soluble polyphenols were not affected by fermentation and improved the antioxidant properties of the yogurt.

Additionally, the extracts can be used as a substrate for making alcoholic beverages or added to confectionery and dairy foods to improve product quality characteristics [6]. Marcillo-Para et al. [64] proposed the encapsulation of apple pomace extracts by spray drying in order to improve the oxidative stability of polyphenols and to enable their controlled release, from the perspective of their use in functional beverages and nutraceutical formulations. Lately, researchers are investigating apple pomace extracts for their ability to naturally deter pests [18].

### 3.4. Simultaneous Response Optimization

The optimal extraction conditions to simultaneously obtain the highest content of phenolic compounds, DPPH radical scavenging activity, and soluble solids content from apple pomace extraction in water were E/S = 9.77%, T = 43 °C, and t = 1 h. The response values at optimum were TPC = 270 mg GAE/L, RSA = 0.68 mmol Trolox/L, and SSC = 4%, while desirability at the optimum location was 98.34%. The experimental results were in good agreement with the predicted values (Table 4). Table 5 presents Pearson correlations (*R*) between the three response variables: total phenolic content, DPPH radical scavenging activity, and soluble solids content of the apple pomace extracts.

Statistically significant positive correlations were found between the response variables in our study. The coefficient of correlation between total phenolic content and DPPH radical scavenging activity equals 0.75, indicating a moderately strong relationship between the variables, while a relatively strong relationship (correlation coefficient = 0.92) was found between RSA and soluble solids content. These results demonstrated that, in addition to phenolic compounds, other water-soluble compounds, such as vitamins, also contributed to the antioxidant activity of the extracts. Some degraded polysaccharides formed during sonication, having better antioxidant properties than ordinary polysaccharides coupled with phenolic compounds, could also contribute to the antioxidant activity of the extracts [65].

### 3.5. Phenolic Characterization of Apple Pomace Extracts

Twelve phenolic compounds, including phenolic acids and flavonoids, have been quantified in the apple pomace extracts (Table 6). The profile was dominated by chlorogenic acid, followed by rutin and epicatechin. Based on previous studies, Antonic et al. [9] reported chlorogenic acid as having the greatest concentration in apple pomace together with catechin, epicatechin, and rutin. In another review by Lyu et al. [6], chlorogenic, caffeic, ferulic, and *p*-coumaric acids, as well as epicatechin, are reported as major bioactive compounds from apple pomace. Waldbauer et al. [66] also listed chlorogenic acid, caffeic acid, catechin, epicatechin, rutin, and quercetin glycosides as the most prominent phenolic compounds in fresh apple pomace. Perussello et al. [3] attributed the apple pomace’s strong antioxidant properties to the presence of phenolics such as epicatechin, quercetin, phloretin, chlorogenic acid, caffeic acid, ferulic acid, *p*-coumaric acid, and phloridzin, among others.

Increasing the extraction time from one to five hours led to a significant decrease in the concentration of chlorogenic acid, both at 40 and 55 °C, while for other phenolic compounds (e.g., caffeic acid) an opposite trend was observed. The caffeic acid content also increased as a result of enzyme addition. Kitrytė et al. [30] also reported that caffeic acid content increased in the chokeberry extract due to the pomace treatment with Viscozyme L. Based on these findings, they even proposed to combine high pressure and enzyme-assisted extraction to obtain caffeic acid-enriched fractions from berry pomaces.

Increasing enzyme addition led to increases in the concentration of phenolic compounds both at 40 and 55 °C after one hour of extraction. For example, a significant increase in ferulic acid content was recorded as a result of enzyme addition. However, at 5 h of extraction, the addition of the enzyme caused a decrease in the content of some flavonoids (for example, the rutin content dropped to less than half). In a study on the impact of thermal, high-pressure, and pulsed electric field treatments on the stability and antioxidant activity of apple pomace extracts, Plamada et al. [67] found that these treatments decreased the content of some phenolic compounds such as phloridzin, chlorogenic acid, and epicatechin; however, these changes did not affect the antioxidant activity of the extracts.

## 4. Conclusions

The use of Box–Behnken experimental design combined with the response surface methodology allowed us to study the influence of enzyme/substrate ratio, temperature, and extraction time on the recovery of phenolic compounds and soluble solids from apple pomace powder. The optimal conditions for producing apple pomace extracts with maximum antioxidant activity, total phenolics, and soluble solids using UEAE with Viscozyme L were an enzyme-to-substrate ratio of 9.7%, a temperature of 43 °C, and an extraction time of 1 h. Chlorogenic acid, caffeic acid, rutin, and epicatechin were the predominant polyphenols in the extracts. The results obtained in this study provide valuable information regarding the bioactive potential of apple pomace aqueous extracts. Future research should be directed at assessing the economic feasibility of the recovery process at the industrial scale using various commercial enzyme preparations, as well as the possibilities of using the extracts obtained, as such or in concentrated form, in food and nutraceutical applications.

## Figures and Tables

**Table 1:** Coded and actual levels of the independent variables based on the Box–Behnken design.

Independent variables		Coded values	
	−1	0	+1
		Actual values	
*x*_1_: Enzyme/substrate ratio (%)	0	5	10
*x*_2_: Temperature (°C)	25	40	55
*x*_3_: Extraction time (h)	1	3	5

**Table 2:** Response values (mean ± SD) of the experimental runs acquired for total phenolic content, DPPH radical scavenging activity, and soluble solids content for the actual levels of process parameters (enzyme/substrate ratio, extraction time, and temperature) in RSM.

Process Parameters (Actual Values)			Total Phenolic Content (mg GAE/L)	DPPH Radical Scavenging Activity (mmol Trolox/L)	Soluble Solids Content (%)
Enzyme/Substrate Ratio (%, *v*/*w*)	Temperature (°C)	Extraction Time (h)			
5	55	5	204.27 ± 6.36	0.63 ± 0.02	3.35 ± 0.05
0	40	1	235.18 ± 2.64	0.57 ± 0.03	2.95 ± 0.10
10	40	1	255.64 ± 5.43	0.67 ± 0.02	3.95 ± 0.07
5	40	3	233.36 ± 1.93	0.66 ± 0.04	3.82 ± 0.05
10	40	5	227.91 ± 6.07	0.66 ± 0.01	3.95 ± 0.00
5	25	5	206.55 ± 3.86	0.57 ± 0.00	3.35 ± 0.07
5	40	3	227.91 ± 0.64	0.65 ± 0.02	3.90 ± 0.05
5	40	3	243.82 ± 5.14	0.65 ± 0.03	3.87 ± 0.05
0	40	5	224.27 ± 4.50	0.53 ± 0.02	2.95 ± 0.10
5	25	1	248.36 ± 7.71	0.65 ± 0.00	3.60 ± 0.00
10	55	3	262.00 ± 1.29	0.67 ± 0.02	3.75 ± 0.05
0	25	3	198.82 ± 5.17	0.49 ± 0.03	2.53 ± 0.03
5	55	1	254.73 ± 3.77	0.65 ± 0.03	3.72 ± 0.05
10	25	3	241.55 ± 4.35	0.62 ± 0.02	3.70 ± 0.07
0	55	3	222.15 ± 3.21	0.53 ± 0.03	2.65 ± 0.10

**Table 3:** Coded and actual values of the independent variables used in the Box–Behnken design.

Regression Coefficients	Total Phenolic Content	DPPH Radical Scavenging Activity	Soluble Solids Content
*β*_0_	173.174 * (0.0000)	0.384421 * (0.0000)	0.346296 * (0.0000)
*β*_1_ (enzyme/substrate ratio)	3.65367 * (0.0030)	0.0269583 * (0.0000)	0.274167 * (0.0000)
*β*_2_ (temperature)	2.64315 (0.0432)	0.0103102 * (0.0018)	0.125185 (0.1630)
*β*_3_ (extraction time)	−1.69729 * (0.0014)	−0.0332292 * (0.0031)	0.0458333 * (0.0091)
*β*_12_	−0.0106 (0.8496)	0.00005 (0.6256)	−0.000333333 (0.5022)
*β*_13_	−0.42025 (0.3222)	0.000625 (0.4187)	0.0 (1.0000)
*β*_23_	−0.0719583 (0.6076)	0.000416667 (0.1147)	−0.000833333 (0.4410)
*β*_11_	0.0690083 (0.6930)	−0.00183333 * (0.0000)	−0.0153333 * (0.0000)
*β*_22_	−0.0246602 (0.2128)	−0.000131481 * (0.0013)	−0.00148148 * (0.0000)
*β*_33_	−0.250885 (0.8181)	0.000729167 (0.7150)	−0.00833333 (0.3918)
*p*-value	0.0003	0.0003	0.0003
*R* ^2^	75.8336	92.1611	96.982

* Significance (*p* ˂ 0.05); *p*-values in parentheses.

**Table 4:** Optimization criteria for process parameters and their responses.

	Goal	Lower Limit	Upper Limit	Impact	Solution	Actual Responses	Desirability
*x*_1_: Enzyme/substrate ratio (%)	In range	0	10	3	9.777	-	-
*x*_2_: Temperature (°C)	In range	25	55	3	43.073	-	-
*x*_3_: Extraction time (h)	In range	1	5	3	1.000	-	-
Total phenolic content (mg GAE/L)	Maximize	193.82	262.91	3	269.973	269.678	98.09
DPPH radical scavenging activity (mmol Trolox/L)	Maximize	0.49	0.69	3	0.684	0.692	97.53
Soluble solids content (%)	Maximize	2.5	4.0	3	4.0658	4.114	99.37

**Table 5:** Pearson correlations (*R*) between total phenolic content, DPPH radical scavenging activity, and soluble solids content in the apple pomace extracts.

	Total Phenolic Content	DPPH Radical Scavenging Activity	Soluble Solids Content
Total phenolic content	1	0.84979 * (0.0001)	0.591497 * (0.0006)
DPPH radical scavenging activity		1	0.921153 * (0.0000)
Soluble solids content			1

* Significant correlations at the 0.001 level (*p*-values in parentheses).

**Table 6:** Phenolic compounds (mg/L) quantified by HPLC-DAD in the apple pomace extracts obtained in selected UEAE conditions.

Phenolic Compound	APE 0-40-1	APE 0-40-5	APE 10-40-1	APE 10-40-5	APE 5-55-1	APE 5-55-5
Vanillic acid	0.34 ± 0.02 ^cd^	0.39 ± 0.02 ^b^	0.44 ± 0.02 ^a^	0.31 ± 0.01 ^d^	0.35 ± 0.02 ^c^	0.39 ± 0.01 ^b^
Rutin	1.42 ± 0.05 ^d^	3.50 ± 0.22 ^a^	1.78 ± 0.08 ^c^	1.59 ± 0.06 ^cd^	1.58 ± 0.07 ^cd^	2.37 ± 0.11 ^b^
Quercetin	0.08 ± 0.01 ^d^	0.03 ± 0.00 ^e^	0.19 ± 0.01 ^b^	0.14 ± 0.01 ^cd^	0.31 ± 0.02 ^a^	0.33 ± 0.02 ^a^
Gallic acid	0.06 ± 0.01 ^c^	0.15 ± 0.02 ^a^	0.05 ± 0.01 ^c^	0.10 ± 0.01 ^b^	0.04 ± 0.00 ^c^	0.10 ± 0.01 ^b^
Catechin hydrate	0.37 ± 0.02 ^c^	0.56 ± 0.04 ^a^	0.27 ± 0.03 ^d^	0.44 ± 0.03 ^b^	0.47 ± 0.03 ^b^	0.49 ± 0.03 ^b^
Syringic acid	0.08 ± 0.01 ^d^	0.05 ± 0.01 ^e^	0.12 ± 0.01 ^c^	0.20 ± 0.02 ^b^	0.03 ± 0.00 ^e^	0.32 ± 0.02 ^a^
Epicatechin	1.61 ± 0.08 ^c^	1.08 ± 0.05 ^d^	2.17 ± 0.13 ^a^	1.92 ± 0.08 ^b^	1.23 ± 0.07 ^d^	1.75 ± 0.11 ^c^
*Trans* cinnamic acid	0.02 ± 0.00 ^b^	0.01 ± 0.00 ^b^	0.06 ± 0.01 ^a^	0.06 ± 0.01 ^a^	0.05 ± 0.01 ^a^	0.05 ± 0.01 ^a^
Chlorogenic acid	16.02 ± 0.31 ^c^	11.81 ± 0.37 ^e^	23.71 ± 0.67 ^a^	15.18 ± 0.45 ^d^	20.41 ± 0.57 ^b^	8.57 ± 0.23 ^f^
Caffeic acid	1.34 ± 0.08 ^e^	2.33 ± 0.02 ^c^	1.58 ± 0.07 ^d^	3.32 ± 0.13 ^a^	1.25 ± 0.05 ^e^	2.85 ± 0.12 ^b^
Coumaric acid	0.51 ± 0.03 ^e^	0.06 ± 0.02 ^f^	0.60 ± 0.02 ^d^	0.92 ± 0.04 ^a^	0.51 ± 0.02 ^e^	0.84 ± 0.03 ^b^
Ferulic acid	0.08 ± 0.01 ^c^	0.11 ± 0.01 ^d^	0.78 ± 0.01 ^a^	0.75 ± 0.04 ^a^	0.37 ± 0.01 ^b^	0.27 ± 0.02 ^b^
Total	91.30 ± 0.63 ^d^	80.76 ± 0.78 ^e^	125.71 ± 1.07 ^a^	98.32 ± 0.89 ^c^	105.95 ± 0.87 ^b^	73.21 ± 0.72 ^f^

Distinct superscript letters represent statistically significant differences (*p* < 0.05) among the apple pomace extracts. APE 0-40-1—apple pomace extract made at E/S = 0%, T = 40 °C, t = 1 h; APE 0-40-5—apple pomace extract made at E/S = 0%, T = 40 °C, t = 5 h; APE 10-40-1—apple pomace extract made at E/S = 10%, T = 40 °C, t = 1 h; APE 10-40-1—apple pomace extract made at E/S = 10%, T = 40 °C, t = 5 h; APE 5-55-1—apple pomace extract made at E/S = 5%, T = 55 °C, t = 1 h; APE 5-55-5—apple pomace extract made at E/S = 5%, T = 55 °C, t = 5 h.

## Data Availability

The original contributions presented in this study are included in the article. Further inquiries can be directed to the corresponding author.
